# A BODY-MIND-SPIRIT CAREGIVER SELF-MANAGEMENT PROGRAM FOR CHINESE AMERICANS: PROGRAM DESIGN

**DOI:** 10.1093/geroni/igz038.2011

**Published:** 2019-11-08

**Authors:** Mandong Liu, Jinli Wu, Geng Zhang, Iris Chi

**Affiliations:** University of Southern California, Los Angeles, California, United States

## Abstract

The aging society presents a growing demand for formal and informal caregivers. Literature review reveals that cultural practices and immigration challenges compound the caregiving burden in Chinese American caregivers. The team designed a culturally sensitive and linguistically appropriate training program targeting the overall well-being of Chinese caregivers. The intervention pivots on the Body-Mind-Sprit model, a health model rooted in Chinese traditional philosophies and integrating three components into an inseparable unity. We designed the in-person training to be four 3-hour sessions and incorporated modalities, which include mini lectures, skill training, discussion, exercise videos, homework activities, and a website. We pilot tested the intervention among 11 Chinese caregivers, followed by two focus group discussions and formative evaluation. Participants provided positive feedback in program evaluation survey, while analysis of focus group revealed three major topics: program benefits, barriers to receiving training and applying its content, and suggestions.

